# Collaboration Networks from a Large CV Database: Dynamics, Topology and Bonus Impact

**DOI:** 10.1371/journal.pone.0090537

**Published:** 2014-03-06

**Authors:** Eduardo B. Araújo, André A. Moreira, Vasco Furtado, Tarcisio H. C. Pequeno, José S. Andrade, Jr

**Affiliations:** 1 Departamento de Física, Universidade Federal do Ceará, Ceará, Brazil; 2 Núcleo de Aplicação em Tecnologia da Informação, Universidade de Fortaleza, Ceará, Brazil; Université de Lausanne, Switzerland

## Abstract

Understanding the dynamics of research production and collaboration may reveal better strategies for scientific careers, academic institutions, and funding agencies. Here we propose the use of a large and multidisciplinary database of scientific curricula in Brazil, namely, the Lattes Platform, to study patterns of scientific production and collaboration. Detailed information about publications and researchers is available in this database. Individual curricula are submitted by the researchers themselves so that coauthorship is unambiguous. Researchers can be evaluated by scientific productivity, geographical location and field of expertise. Our results show that the collaboration network is growing exponentially for the last three decades, with a distribution of number of collaborators per researcher that approaches a power-law as the network gets older. Moreover, both the distributions of number of collaborators and production per researcher obey power-law behaviors, regardless of the geographical location or field, suggesting that the same universal mechanism might be responsible for network growth and productivity. We also show that the collaboration network under investigation displays a typical assortative mixing behavior, where teeming researchers (*i.e.*, with high degree) tend to collaborate with others alike.

## Introduction

Nowadays, scientific collaboration is understood as extremely valuable, as it integrates skills, knowledge, apparatus and resources, allows division of labor and the study of more difficult problems, including interdisciplinary ones. It also brings recognition and visibility and increases the network of contacts of the researchers involved [1]–[3]. Scientific collaboration is strongly correlated with production measured by publication output and other indexes in Scientometrics [4]–[6], which has substantially contributed to raise the interest of the scientific community in studying itself over the last decades [2], [4], [7]–[10]. More recently, due to the fast growth and enormous development of the complex network science [11]–[22] the subject of scientific collaboration has been extensively studied under the framework of rather powerful and universal paradigms [23]–[29].

The Internet and the fact that traveling became substantially less costly have facilitated international collaborations. Still, geographical constraints affect the dynamics of research [30]–[32]. Different countries have different funding policies and this impacts the publication outcome, which is correlated to collaboration. For a country to be above the world average number of citations, it must spend more than one hundred thousand US dollars per researcher per year [32]. At the same time, scientists with more investment in their research projects collaborate more [33].

The social nature of collaboration [2], [34] might be the cause for the big disparity in production and number of collaborators [35]. Inequalities in income (Pareto distribution [36]) and movie co-appearance [37] are examples of social distributions, characterized by a power-law profile. For scientific collaborations, such distributions also appear, as demonstrated by Lotka [38], from the analysis of two empirical sets of publications data in natural sciences.

Although in Lotka's analysis [38] only the senior authorship has been considered, the obtained power-law was shown to be consistent with empirical bibliometric data taking all authors into account [39]. The so called Lotka's Law therefore seems to be valid even in different fields than those originally considered [39], [40]. It is also worth noting that highly prolific authors were excluded in Lotka's procedure due to the limited number of persons in the samples. These teeming researchers might lie outside the pure power-law distribution. Considering that engaging in collaboration is a time consuming activity, the number of collaborators can not be arbitrarily large, i.e., must be somehow limited. An exponential cutoff has then been suggested as a correction to fit the distribution of productivity [27]. Measuring the distributions of citations by city or country, a power-law distribution also arises [32], which indicates the presence of self-similarity in the science system [41].

Nonetheless, the definition of research collaboration is problematic due to the subjective understanding of its essential ingredients [2], [3]. This can be avoided by considering as scientific collaboration a research which resulted in a coauthored scientific paper. This approach, although traditional, is not free of criticism as there are fruitful and relevant collaborations which do not necessarily involve a publication. Notwithstanding, there is evidence that division of labor of theoretical or experimental work is usually rewarded with a coauthorship [3]. Also, analysing coauthorship makes it feasible to study collaboration of a greater number of researchers as compared by interviewing each individual.

Despite the numerous studies about scientific production, citations and collaborations found in the literature, it is difficult to compare these variables as the databases used in these studies are usually unrelated. Another problem is the small number of samples, due to a low number of respondents in questionnaires or data used only from a specific journal. To analyse the big picture is paramount to work with a dense information database. Here, we used data from Lattes Platform (http://lattes.cnpq.br), an online database maintained by CNPq (National Council of Technological and Scientific Development), a government agency that finances scientific research in Brazil. It contains the curricula of almost all researchers in Brazil and their collaborators abroad, as well as information concerning their research groups. The Lattes Curriculum became the standard national scientific curriculum in Brazil, and compulsory for those requiring financial support from the Brazilian government. The curricula present detailed information concerning the researcher, including, but not limited to, full name, gender, professional address, academic titles, field of expertise and list of papers. Researchers are classified in 8 major fields: Agricultural Sciences (Agr), Applied Social Sciences (Soc), Biological Sciences (Bio), Exact and Earth Sciences (Exa), Humanities (Hum), Health Sciences (Hea), Engineering (Eng), Linguistics and Arts (Lin), and Others (Oth). Most information in the curriculum are provided by the researcher themselves, for example, their list of publications.

By using this database, we may overcome some of the limitations found by other authors [23], [24]. Due to the lack of individual information of the researcher, the problem of author name disambiguation [24], [42] becomes relevant, when, for example, two or more authors share initials and surnames. This is not the case with the Lattes Platform, where coauthorship is unambiguous. Researchers themselves update their curricula with detailed information about their publications and professional activity. As a consequence, this type of data allows us to study scientific production and collaborations of individual researchers and correlations between fields of expertise.

## Methods

The collaboration networks are build based on data of approximately 2.7 million curricula downloaded in June 2012 from the Lattes Platform website. Files are parsed to extract the name of the researcher, professional address and authored papers published in periodicals (including title, year and number of coauthors in the paper).

Due to possible typographical errors [43], an approximate string matching is used to compare paper titles. We use Damereau-Levenshtein distance [44] as the metric and compare papers of the same year and with the same number of authors starting with the same letter. Papers differing by 10% or less of the maximum distance are considered to be the same paper.

From the string matching results, we build a unweighted bipartite network 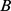, with node classes 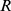 and 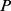, representing researchers and papers, respectively. A researcher 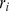 in 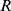 is connected to a paper 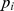 in 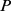 if 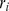 is identified as one of the authors of 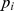 in the former procedure. Nodes store the information parsed previously: 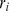 contains gender, fields of expertise, professional address and scholarships information while 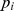 contains title, number of coauthors and year.

**Figure 1:**
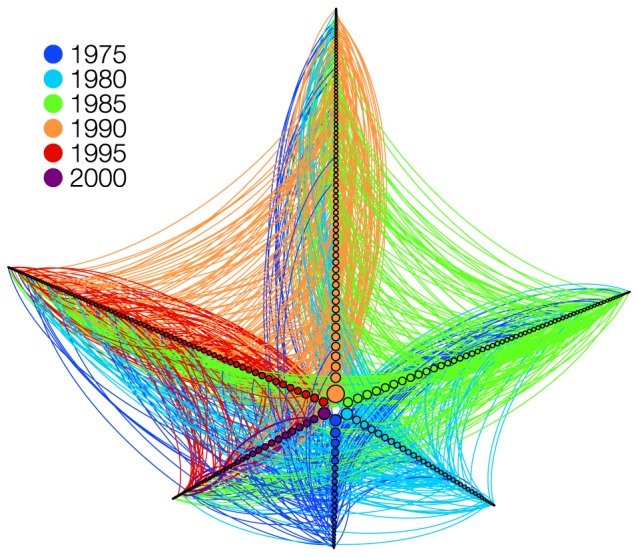
Sample network extracted from the collected data. We show links between researchers (nodes) who were granted a scholarship and working in fields of Medicine in the state of São Paulo. Node size is proportional to the degree of the researcher in the whole database. Researchers were grouped according to the year of their first published paper. The first cohort (dark blue) comprises all researchers who published their first paper before 1975. Each subsequent one, in counterclockwise direction, comprises researchers who published within 5 years from the previous one, up to 2000. The edges are directed, colored according to the most senior.

We focus our study on a projection of the bipartite network onto 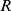. There are many ways to accomplish this [45], the simplest being to project 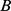 onto an unweighted undirected network, with researchers 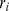 and 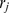 connected if both are connected to a paper 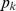 in 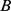. We used this method to construct a cumulative network containing collaborations of all researchers in the database, the Total Collaboration Network (TCN). One should note that, with this database, we are not limited to the simple projecting scheme, since information on researchers and papers can be used in the projection. In order to illustrate this procedure, we show in Fig. 1 a network constructed only with researchers working on fields of Medicine in the state of São Paulo and with a grant from the Brazilian government. We did the projection in such way that the edges are directed, pointing to the researcher with the earliest date of publication of a paper. Unless noted otherwise, all the network projections analysed in this work are unweighted and undirected.

The parameter for the exponential functions were estimated by logarithmic transformation and subsequent linear regression. For the power-law with exponential cutoff distributions, 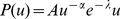, the 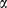 parameters were initially estimated by numerically maximizing the corresponding log-likelihood function [46]. The values for the lower bounds of the modeled behavior, 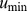, were estimated from the corresponding Hill plot [46]. Subsequently, the 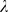 parameters were estimated using the Levenberg-Marquardt Algorithm (LMA) with the previously estimated value of 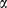 and 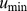.

For power-law functions, we perform a logarithmic transformation followed by linear regression to calculate the power-law exponent.

## Results and Discussion

TCN includes 275,061 researchers, with 90.4% belonging to the largest component. The total number of identified papers written in collaboration is 623,984, the number of collaborations is 1,095,871 and the network comprises all 8 major fields used by the Brazilian agency CNPq to classify researchers.

**Figure 2:**
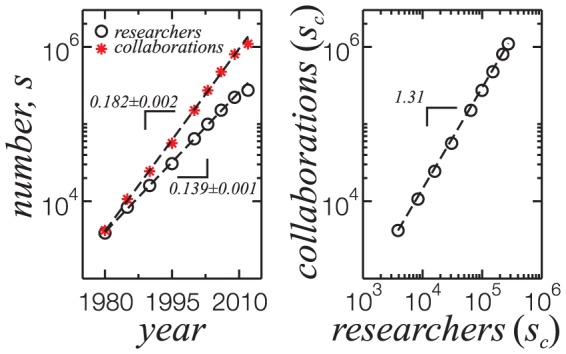
Left: Number of researchers with published papers (black circles) and collaborations between them (red stars) present in the cumulative collaboration network. Dashed lines are exponential fits in the form 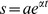 up to 2009, seen as straight lines in the linear-log plot. The coefficient 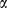 is shown in the picture for each curve. Deviations of the 2012 data points from the exponential fit are due to the early acquisition of the curricula, in June of 2012. Right: Superlinear scaling of the number of collaborations with the number of researchers. Dashed line is a power-law curve with exponent 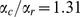.

The extracted papers have publication date extending for several decades, the oldest paper in collaboration being from 1949. By analysing the growth of the network, we show in Fig. 2 (left) that the number of researchers (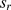) as well as collaborations (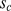) grew exponentially in the last three decades, 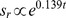 and 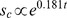, with 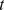 in years. We also show that the number of collaborations increases superlinearly with the number of researchers in the network. This accelerated growth has been observed in collaboration networks [23], [47] and other types of empirical networks [48]. More recently, it was shown that the number of social contacts and total communication also scales superlinearly with city population size [49].

**Figure 3:**
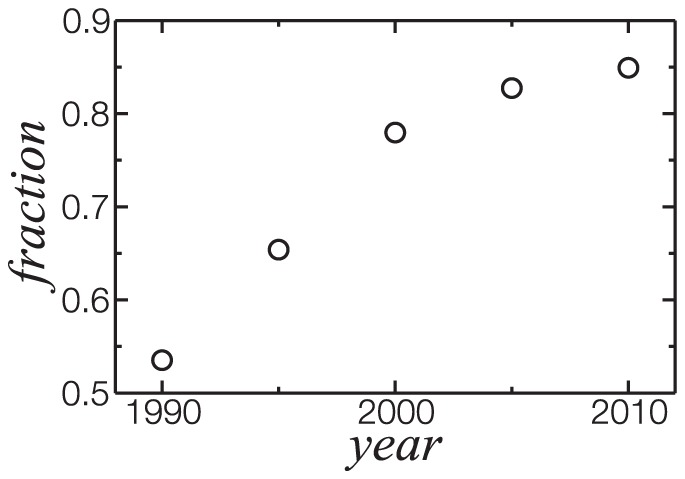
Evolution of the largest component. Data points represent the fraction of researchers present in the largest component for a five year time window centered in the respective year. More than 80% of the researchers engaged in collaborations in the last 5 years are in the largest component. They represent 61% of the researchers in TCN.

To analyse the evolution of the largest component, we construct networks with a limited time window spanning five years centered in 1990, 1995, 2000, 2005 and 2010. This was accomplished projecting the bipartite network linking researchers connected to papers published only within the respective time window. Fig. 3 shows an increase in the largest component fraction over years, with a fraction 84.9% of researchers in the last data point. For this time window, we obtained the fraction of each field, shown on Table 1, indicating that fields are mixed in the largest component in the same proportion as in the complete network. The fact that more than 80% of the network is connected together with the field distribution is an interesting sign, which indicates that discoveries from a field can spread in the communities through interdisciplinary collaborations. As this last network is a subgraph of TCN, most of the links in latter were active in the last 5 years.

**Table 1:** Fraction of fields in the last 5 years.

Field	fraction in largest component	fraction in the network
Agr	13.9%	12.2%
Bio	18.0%	15.8%
Hea	26.3%	24.1%
Exa	13.0%	12.3%
Hum	5.9%	8.9%
Soc	5.1%	7.3%
Eng	6.5%	6.5%
Lin	0.5%	1.8%

The network was constructed by projecting the bipartite network onto a network containing only reseachers connected if they share a paper published in the last 5 years. Sum of fractions is not 100% because the field information is not available for all researchers.

A commendable initiative of the Brazilian government is to award scholarships to distinguished researchers among their peers. Doctorates may apply for several levels of scholarship. Applications are judged by a committee based on requestor's project, scientific contributions, participation as a journal editor, among other criteria. These scholarships correspond to a bonus payment in addition to their base salary. The scholarship information is included in the CV by CNPq, not by the researcher, and we obtain the list of researchers awarded when parsing their curricula. For comparison with the TCN, we built a collaboration network with only these researchers, projecting the bipartite network 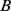 onto 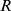 connecting only awarded researchers with shared papers on 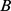, which we call Scholarship Collaboration Network (SCN). SCN is therefore a subgraph of TCN. In Table 2 we show the basic statistical properties of TCN and SCN.

**Table 2:** Statistics for the networks studied in this work.

	TCN	SCN
Number of researchers ( 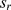 )	275,061	12,302
Number of edges ( 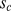 )	1,095,871	134,186
Total number of papers	623,984	129,699
Average researchers per paper	4.51	5.26
Average papers per author ( 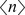 )	11.1	61.4
Average number of collaborators ( 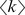 )	8.0	38.1
Largest component fraction	90.4%	94.6%
Clustering coefficient ( 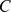 )	0.465	0.266
Assortativity coefficient ( 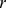 )	0.094	0.230

The clustering coefficient [11], 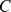, measures the probability that two collaborators of a given researcher have papers in common (forming a triangle in the graph). Social networks are known to have high degree of clustering [14], which can be explained in terms of a hierarchical structure [15]. Here both networks display a high clustering coefficient but the average value for SCN is about half of TCN. This difference reflects the higher position in the research groups of the researchers with scholarship. They are more likely to have contacts in other research groups, which means being less clustered.

**Figure 4:**
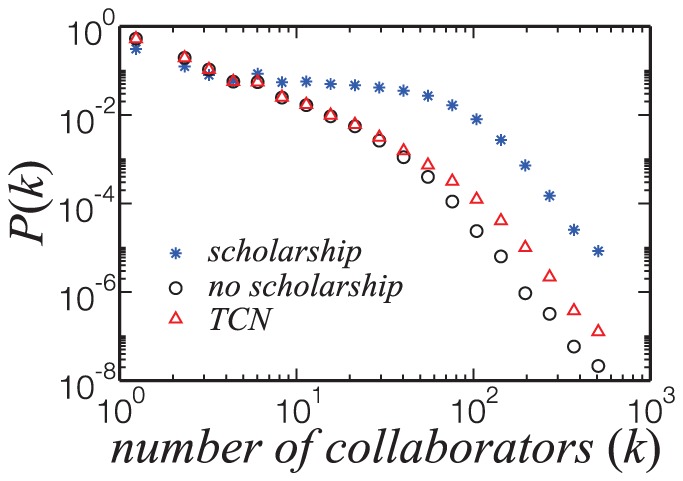
Normalized distribution of the number of collaborators (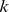) of researchers with scholarship (blue stars), without (black circles) and for the TCN (red triangles). The distribution for researchers with scholarship decreases slowly up to one hundred collaborators, although most of them still have a small number of collaborators. The higher proportion of researchers with high 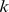 might reflect the CNPq policy of considering the proponent's participation in research groups, international immersion and human resources development to grant the scholarship.

A relevant question which naturally arises is how the scientific productivity and collaboration statistics of researchers awarded with scholarships differ from regular researchers. Studying our database, we find that researchers in the SCN represent less than 5% of the researchers in the TCN but contribute with 20% of the production. They are in average more than five times more productive, as measured by publication output. Also, SCN is more cohesive than TCN, as measured by the size of the giant component. To determine whether these characteristics are cause or consequence of their scholarship is not our aim, but previous research on collaborations strategies indicate that those with higher grants are more likely to have more collaborators [33]. The degree distributions shown in Fig. 4 clearly corroborate this difference between groups.

**Figure 5:**
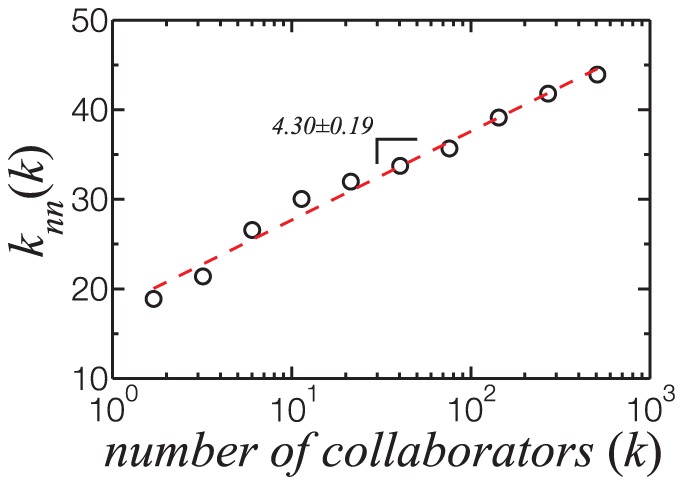
Variation of the average nearest-neighbor degree (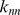) with 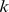. Being an increasing function of 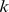, the network displays assortative mixing. Researchers with high 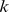 are more likely to collaborate with other well connected researchers. This tendency, however, increases logarithmically with 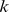, as indicated by the regression fit (dashed line).

The assortativity coefficient [13], 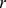, measures the correlation between degrees of nodes at either ends of an edge. Networks with 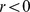 are said to display disassortative mixing, while 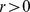 means assortative mixing. Social networks, including collaborations networks, are known to display assortative mixing [13], [16]. Another way of looking at the assortative properties of a network is through the average nearest-neighbor degree, 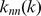 [17], where 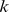 is the number of collaborators of a researcher. This measures how well connected the collaborators of a researcher are. If 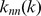 is an increasing function, then researchers with high 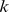 collaborate with other well-connected researchers, and the network displays assortative mixing. We show in Fig. 5 that this occurs in TCN, and that 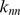 increases logarithmically with 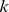. Assuming that researchers with a high number of collaborators are positioned in the top of the academic hierarchy, we can infer from Fig. 5 that prominent researchers and group leaders collaborate more among themselves. Nonetheless, 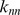 does not grow fast but logarithmically, as researchers growing in importance absorb the influx of new actors in the network.

**Figure 6:**
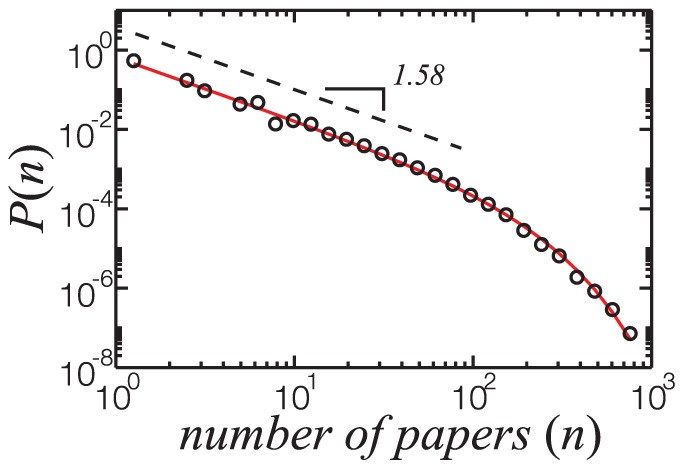
Distribution of scientific production of researchers belonging to the TCN group. The solid red line is the best fit to the data points of a power-law with exponential cutoff, 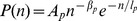, where 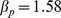 and 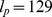. The dashed black line is a power-law with exponent 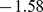.

It is inviting to verify if the production of researchers on Lattes Platform obeys Lotka's Law. As shown in Fig. 6, the distribution of scientific production (in number of papers, 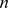) obeys a power-law with exponential cutoff, 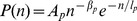, with exponent 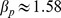 and characteristic cutoff length 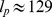.

**Figure 7:**
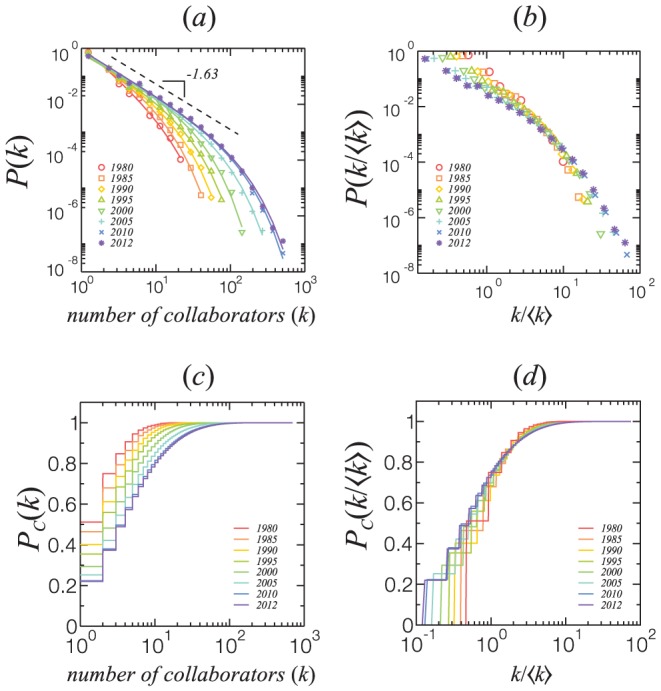
(a) Time evolution of the distribution of the number of collaborators in the TCN. (b) Rescaling the distribution in (a) by the relative number of collaborators for each year shows a collapse onto a single curve. We also show the respective cumulative distributions in (c) and (d). As the network ages, the fraction of researchers with high 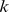 increases (c), but the evolution of the network shows that the distribution is constrained to the average production (d).

With this database, we can study the time evolution of the cumulative collaboration network by analysing different groups of papers that have been published within a specific range of years. We show in Fig. 7 (a) the evolution of the distribution of the number of collaborators in TCN, from 1980 to 2012. We show in Fig. 7 (b) a rescaling of these curves by the relative number of collaborators for each year, collapsing onto a single curve. Figs. 7 (c) and (d) show the respective cumulative distributions. Although the cumulative distribution varies with year, with the increase of highly connect researchers, this distribution is constrained to the average number of collaborators of TCN (d).

**Figure 8:**
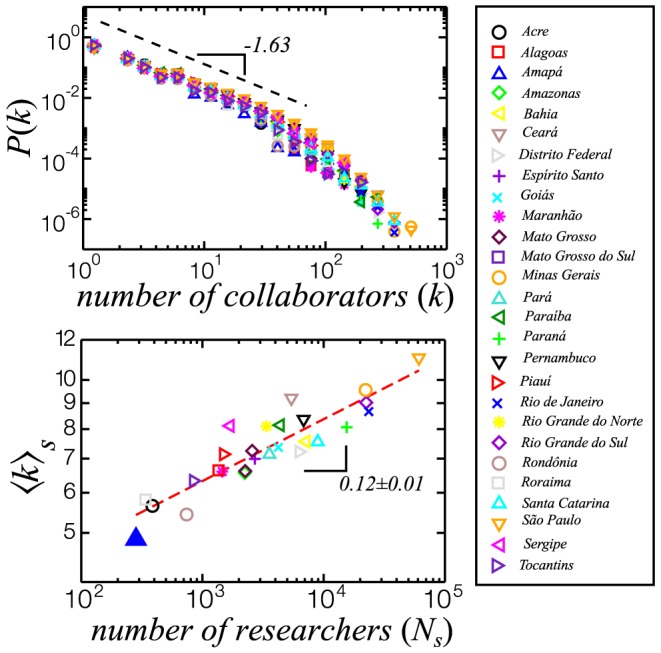
Top: Distribution of number of collaborators in the TCN for the 26 Brazilian states and the Federal District. The distributions display the same behavior as the TCN (Fig. 7). The dashed line is a power-law with exponent 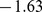. Bottom: the average number of collaborators versus the number of researchers in each state. The circles correspond to the results for 26 Brazilian states and the Federal District. The dashed line is the best fit obtained by linear regression of the data to a power-law 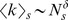 in logatirhmic scale, with exponent 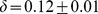.

We can use the professional address information included in the curricula to study the differences of collaboration profile due to geographical location. As shown in Fig. 8 (top), the overlap of the degree distributions for the TCN at each of the 26 states of Brazil and Brasília, the Federal District, suggests universality in the collaboration mechanism. The geographical location of the researcher, while not changing the shape of the distribution, is correlated with the spectrum of the number of collaborators. Recent allometric studies show that a large number of urban indicators (e.g., R&D employment, total wages, GDP, gasoline sales, length of electrical cables) scale as a power-law of population of the city [50]. In Fig. 8 (bottom) we show that the average number of collaborators per researcher in the Brazilian states 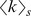 generally increases with their number of researchers as a power-law, 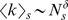 with an exponent 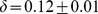.

**Figure 9:**
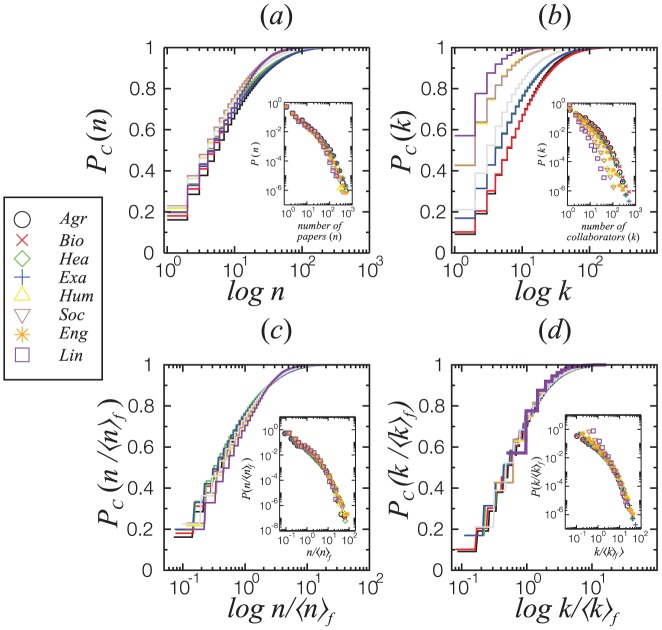
Cumulative distributions 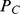 of the number of papers published per researcher 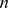 (a) and number of collaborators (b) for each of the 8 major fields. The respective distributions for the rescaled data are shown on (c) and (d). Lines represent different fields, colored according to the symbol in the legend. Scientists working on social sciences and related fields (Lin, Soc and Hum) are less likely to have published more than one hundred papers than others. They also are less likely to have more than one hundred collaborators. Considering the average publication count 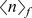 and average number of collaborations 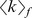 in each field, all the curves collapse to a single universal behavior. The insets show the respective (non-cumulative) distributions.

Finally, the way researchers from different fields collaborate can also be investigated with the data downloaded from the Lattes platform. Fig. 9(a) and (b) show that the cumulative distributions of researcher productivity 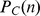 as well as their corresponding degree distributions 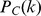, respectively, can be rather different for distinct fields. However, since different fields are known to have different levels of productivity [51], by rescaling 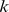 and 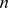 to the corresponding average values of the field (see Table 3), 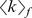 and 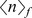, both 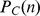 and 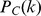 distributions collapse to single universal curves, as depicted in Figs. 9(c) and (d), respectively.

**Table 3:** Statistics for researchers working on the 8 major fields associated with the TCN.

	Number of researchers ( 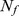 )	Researchers with scholarship ( 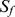 )	Average number of papers per researcher ( 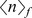 )	Average number of collaborators ( 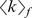 )
Agr	31812	1692	13.9	11.7
Bio	39767	2605	13.1	12.5
Hea	67561	1511	12.6	9.08
Exa	33310	3273	13.5	9.16
Hum	26263	1324	8.90	3.21
Soc	20806	742	8.66	3.23
Eng	18365	1841	10.2	6.37
Lin	5202	300	9.09	2.06

## Conclusions

In summary, we have used the Lattes Platform, which contains detailed and unambiguous data of approximately 2.7 million curricula of researchers, as a database for analysing research collaboration in Brazil. It has the advantage of displaying individual curricula, allowing us to study collaborations in a mix of a paper-based approach and questionnaire data.

We therefore built collaboration networks including all researchers data from Lattes Platform as June 2012, and found that the network has grown exponentially for the last three decades. The calculated values of the assortativity coefficient and the average nearest-neighbor degree indicate that the networks display assortative mixing, where researchers having high 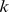 collaborate with others alike. Our results show that these teeming researchers are more likely to have a scholarship and to produce more papers than researchers with low 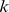. The distribution 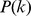 is also approaching a power-law as the network gets older.

Finally, we confirmed the validity of Lotka's Law for researchers working on different states of Brazil and found substantial correlations between 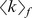 and 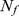. Lotka's Law is shown to be valid for different fields: indeed, 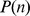 and 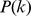 follow an universal behavior.
